# Retracted: MicroRNA-124 Regulates the Proliferation of Colorectal Cancer Cells by Targeting iASPP

**DOI:** 10.1155/2023/9847281

**Published:** 2023-01-04

**Authors:** BioMed Research International

*BioMed Research International* has retracted the article titled “MicroRNA-124 Regulates the Proliferation of Colorectal Cancer Cells by Targeting iASPP” [1] due to extensive similarity to a published article that was not cited, which also studied microRNA-124 in colorectal cancer [2].

Additional concerns were raised regarding the verification of nucleotide sequences used in the study [3], however, the authors were not able to provide a satisfactory response to the journal. The editorial board have determined that retraction is required due to the extensive text overlap. The authors do not agree to the retraction.
